# Generation of functional scFv intrabody to abate the expression of CD147 surface molecule of 293A cells

**DOI:** 10.1186/1472-6750-8-5

**Published:** 2008-01-29

**Authors:** Khajornsak Tragoolpua, Nutjeera Intasai, Watchara Kasinrerk, Sabine Mai, Yuan Yuan, Chatchai Tayapiwatana

**Affiliations:** 1 Division of Clinical Immunology, Department of Medical Technology, Faculty of Associated Medical Sciences, Chiang Mai University, Chiang Mai, 50200, Thailand; 2 Division of Clinical Microscopy, Department of Medical Technology, Faculty of Associated Medical Sciences, Chiang Mai University, Chiang Mai, 50200, Thailand; 3 Biomedical Technology Research Unit, National Center for Genetic Engineering and Biotechnology, National Science and Technology Development Agency at the Faculty of Associated Medical Sciences, Chiang Mai University, Chiang Mai, 50200, Thailand; 4 Department of Physiology, Manitoba Institute of Cell Biology, CancerCare Manitoba, University of Manitoba, Winnipeg R3E 0V9, Canada; 5 Department of Molecular Biology and the Skaggs Institute for Chemical Biology, The Scripps Research Institute, La Jolla, California 92037, USA; 6 Millipore Bioscience Division, 28820 Single Oak Drive, Temecula, CA 92590, USA; 7 BioMedical Engineering Center, Chiang Mai University, Chiang Mai, 50200, Thailand

## Abstract

**Background:**

Expression of intracellular antibodies (intrabodies) has become a broadly applicable technology for generation of phenotypic knockouts *in vivo*. The method uses surface depletion of cellular membrane proteins to examine their biological function. In this study, we used this strategy to block the transport of cell surface molecule CD147 to the cell membrane. Phage display technology was introduced to generate the functional antibody fragment to CD147, and we subsequently constructed a CD147-specific scFv that was expressed intracellularly and retained in the endoplasmic reticulum by adenoviral gene transfer.

**Results:**

The recombinant antibody fragments, Fab and scFv, of the murine monoclonal antibody (clone M6-1B9) reacted specifically to CD147 by indirect enzyme-linked immunosorbent assays (ELISA) using a recombinant CD147-BCCP as a target. This indicated that the Fab- and scFv-M6-1B9 displaying on phage surfaces were correctly folded and functionally active. We subsequently constructed a CD147-specific scFv, scFv-M6-1B9-intrabody, in 293A cells. The expression of CD147 on 293A cell surface was monitored at 36 h after transduction by flow cytometry and demonstrated remarkable reduction. Colocalization of scFv-M6-1B9 intrabody with CD147 in the ER network was depicted using a 3D deconvolution microscopy system.

**Conclusion:**

The results suggest that our approach can generate antibody fragments suitable for decreasing the expression of CD147 on 293A cells. This study represents a step toward understanding the role of the cell surface protein, CD147.

## Background

CD147 is a 50–60 kDa transmembrane glycoprotein. The molecule has an external domain consisting of two regions exhibiting the features of the immunoglobulin superfamily [1-3]. CD147 is widely expressed in both hematopoietic and non-hematopoietic cells and tissues [4-7]. However, the molecule is strongly expressed on various cancer cells, thymocytes and activated T lymphocytes [3,6,8-12]. CD147 is involved in cellular adhesion [8,13,14], lymphocyte activation [14-16], membrane transport [17-19] and signal transduction [20-23]. In addition, CD147 plays a crucial role in the invasive and metastatic activity of tumor cells [9,24,25]. Inhibition of CD147 cell surface expression may help to elucidate these physiological functions of CD147.

A negative regulatory function for CD147 in T cell regulation has been demonstrated [14-16,26]. Recently, two anti-CD147 mAbs, M6-1E9 and M6-1B9, which react with the membrane-distal Ig domain, have been shown to inhibit OKT3-induced T cell proliferation [14]. Presumably, prevention of cell division is caused by delivery of a negative signal *via *CD147. Another possibility is prevention of CD147 becoming associated with its cell surface partners, which may cooperate in CD3 signaling to generate the complete activation signal. The latter hypothesis may be investigated by blocking the expression of surface CD147.

Intracellularly expressed antibodies (intrabodies) can inhibit protein function in specific cellular compartments [27]. They have the capacity to inhibit the translocation of cell surface molecules from the endoplasmic reticulum (ER) to the cell surface as ER-intrabodies [27-29]. Intrabodies offer an effective alternative to gene-based knockout technology [30]. This technique has several advantages compared to RNA interference (RNAi) technology, since intrabodies possess a much longer active half-life than RNA, are much more specific to their target molecules [31,32] and generally do not disrupt target gene transcription. Moreover, gene knockout and silencing techniques cannot be used to analyze domain functions and post-translationally modified protein functions.

The aim of the present study was to generate an intrabody against CD147 in order to down-regulate the cell surface expression of CD147 and retain this surface molecule inside the cell. Sequences encoding both variable regions of heavy chain (V_H_) and light chain (V_L_) domains against CD147 were cloned from hybridomas producing monoclonal antibody clone M6-1B9. These sequences were joined by a flexible peptide linker sequence, allowing the expression of scFv as a single polypeptide chain. The functional activities of this intrabody, *i.e. *target tracing and capturing, were verified in a human embryonic kidney cell line, 293A, which naturally expresses CD147. This manipulation of cell surface CD147 expression could serve as a basis for the generation of CD147-down regulated cells, and represents a step toward characterizing the role of CD147 in regulation of lymphocyte activation and induction of matrix metalloproteinase production by tumor cells.

## Results

### Construction of a phagemid vector encoding scFv-M6-1B9

**Figure 1:**
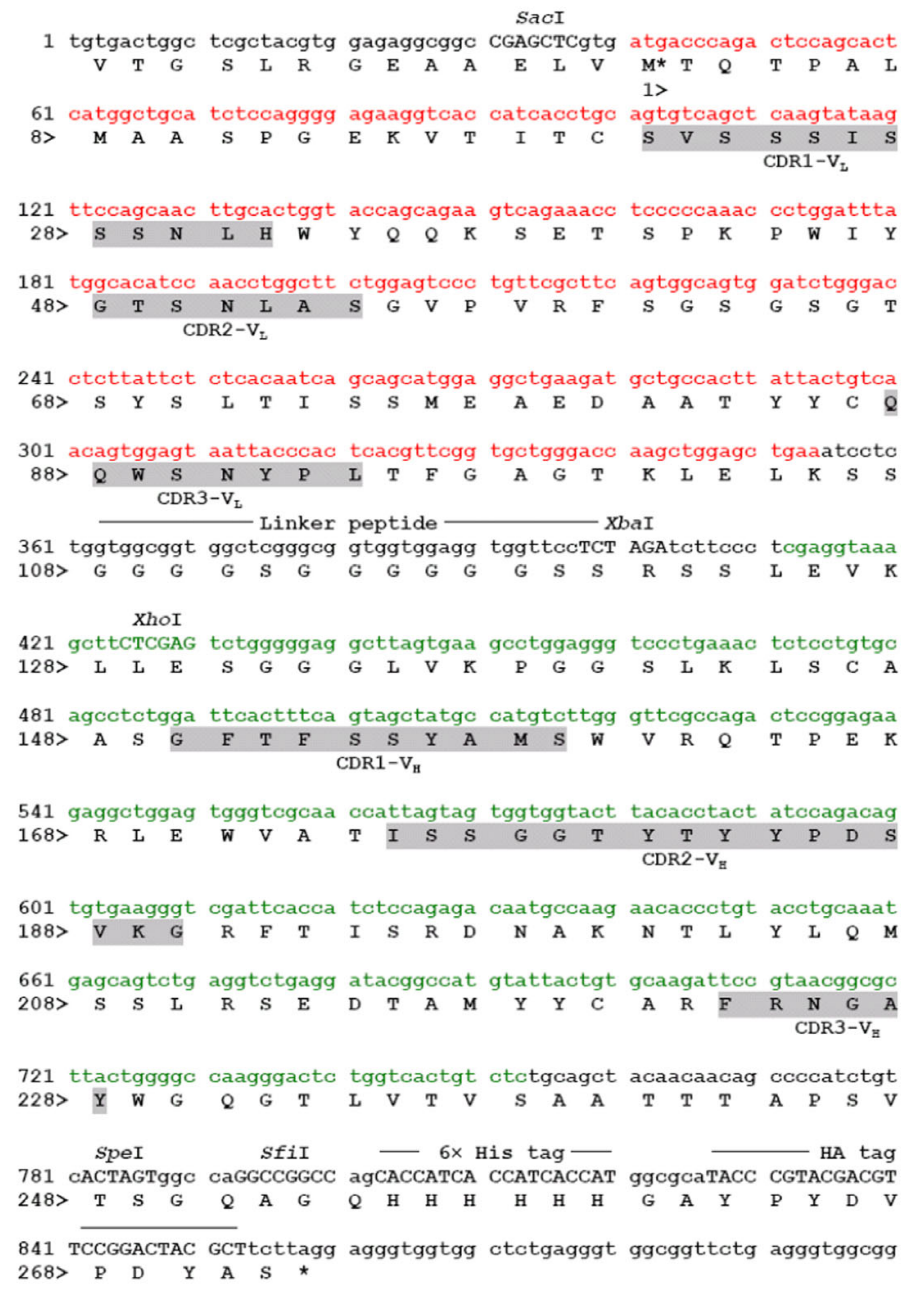
**Nucleotide sequence of cDNA and deduced amino acid sequence of the scFv-M6-1B9**. The cDNA sequence encoding scFv-M6-1B9 was shown. Restriction endonuclease sites, His tags, and HA tag are indicated. The deduced amino acid sequence of scFv-M6-1B9 corresponding to the complementary determining regions (CDRs) in the variable regions of the L (red letters) and H (green letters) chains, which were identified by the Kabat numbering scheme, are indicated by gray boxes. Amino acids were numbered from the initiator methionine (M*). The amber stop codon was shown by an asterisk (*). The details of the CDRs region of scFv-M6-1B9 are shown as CDR1-V_L _C (^24^SV---LH^35^) W, CDR2-V_L _Y (^51^GT---AS ^57^) G, CDR3-V_L _C (^90^QQ---PL^97^) T, CDR1-V_H _S (^153^GF---MS^162^) W, CDR2-V_H _A (^178^IS---KG^193^) R, and CDR3-V_H _R (^226^FR---GAY^231^) W.

The heavy, Fd, and light chain domains of anti-CD147 mAb, M6-1B9 [8], were amplified, subcloned into the expression vector and then named as pCom3H-Fab-M6-1B9. Subsequently, the V_L _and V_H _were amplified from pCom3H-Fab-M6-1B9 and attached by a peptide linker to form the scFv. The amplified product was cloned into phagemid vector pComb3X, named pComb3X-scFv-M6-1B9, and then transformed into *E. coli *TG1. The nucleotide sequence of the inserted fragment, scFv, was obtained (Figure 1). The scFv construct was fused to the carboxy-terminal domain of the minor coat protein, gpIII, and displayed on the surface of phage particles. The deduced amino acid sequences of variable heavy (V_H_) and light (V_L_) chains are listed in Figure 1. The amino acid residues responsible for paratope in CDR regions were subsequently identified *via *the WAM (for Web Antibody Modelling) algorithm [33]. The sequence can be numbered following Kabat's rule [34], in order to assure success in cloning the immunoglobulin variable domains.

### Detection of phage-displayed scFv-M6-1B9

**Figure 2:**
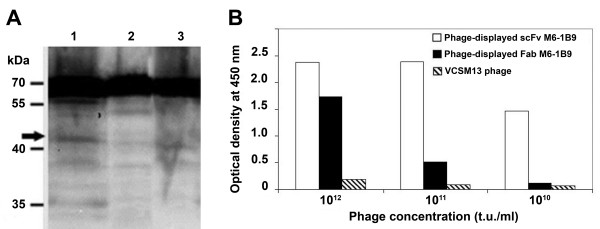
**Verification of antibody phage presenting different formats**. **A **Recombinant phages (10^13 ^t.u./lane) were separated on a reducing 12% SDS-PAGE. The gpIII protein was probed using anti-gpIII mAb. The immunoreactive bands were visualized by chemiluminescence substrate detection system. Lane 1, phage-displayed scFv-M6-1B9; lane 2, phage-displayed Fab-M6-1B9 and lane 3, VCSM13 helper phage. Molecular weight markers in kDa are indicated. Arrow indicates recombinant antibody fragment-gpIII fusion proteins (~47 kDa). **B **CD147-BCCP was captured on wells coated with avidin. Three different concentrations, 10^10 ^– 10^12 ^t.u./ml of phage-displayed scFv-M6-1B9 and phage-displayed Fab-M6-1B9 were added and traced by peroxidase-conjugated anti-M13 phage mAb. VCSM13 helper phage and mAb M6-1B9 specific for CD147 [8] were used as wild-type phage control and antibody control, respectively.

The expression of Fab- and scFv-M6-1B9 on phage particles was assessed by Western immunoblotting. Equal amounts of each recombinant phage were fractionated by SDS-PAGE, blotted, and probed with anti-gpIII mAb. Wild-type gpIII has a calculated molecular mass of approximately 44 kDa. However, a 62 kDa protein detected in Western blots using anti-gpIII specific antibodies has been previously demonstrated [35-37]. The truncated form of gpIII is used in pComb3 vector which has a molecular mass of 18.7 kDa. In case of Fab antibody fusion format in the present study, Fd fragment was fused to truncated gpIII. This resulted in migration of Fd-gpIII fusion at 47 kDa as indicated by arrow (28.7 kDa of Fd fused with 18.7 kDa of truncated gpIII). Likewise, the molecular mass of scFv is also approximately 28.7 kDa which resulted in a molecular weight of 47 kDa of scFv-gpIII fusion protein. Thus, the immunoreactive bands of scFv-M6-1B9- and Fab-M6-1B9-gpIII fusion protein with the approximate molecular weight of 47 kDa were obtained (Figure 2A). Noticeably, the band corresponding to the scFv-gpIII fusion protein was more prominent than the band corresponding to the Fab-M6-1B9-gpIII fusion protein, reflecting the fact that expression of scFv was superior to that of Fab obtained by the phage display technique. Antigen-specific binding of phage presenting the different antibody formats was verified by ELISA using recombinant CD147-biotin carboxyl carrier protein (BCCP) fusion protein as an antigen. The scFv format demonstrated more favorable antigen-binding features than the Fab format (Figure 2B). In contrast, VCSM13 phage prepared from non-transformed TG-1 did not generate the signal against CD147-BCCP antigen. These results suggest that phage presenting different antibody formats of M6-1B9 had been successfully produced and that the scFv version was the better functional antibody fragment.

### Detection and characterization of soluble scFv-M6-1B9 produced in *E. coli*

**Figure 3:**
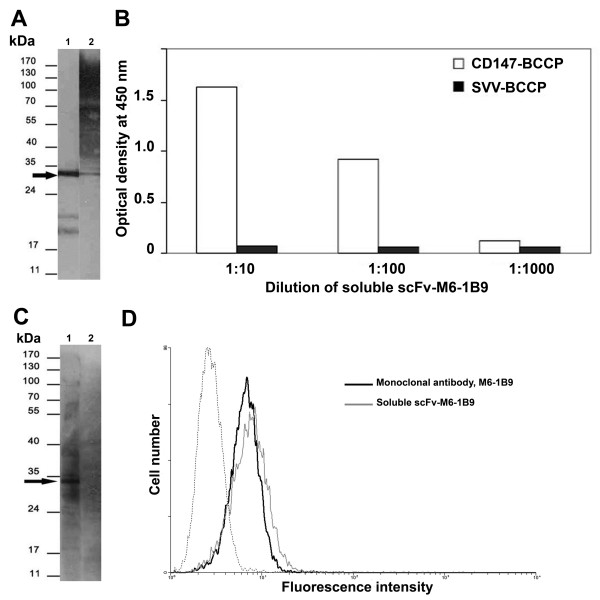
**Detection of soluble scFv**. **A **Soluble scFv-M6-1B9 was separated on 12% SDS-PAGE, electroblotted onto PVDF membrane, and probed with peroxidase-conjugated mAb anti-HA (lane 1) and anti-His mAb (lane 2). The immunoreactive bands were visualized by ECL substrate detection system. The molecular weight is indicated. **B **CD147-BCCP (open columns) or SVV-BCCP (black columns) was captured on the avidin-coated wells. Soluble scFv-M6-1B9 was subsequently added and the bound scFv was detected by peroxidase-conjugated mAb anti-HA. **C **CD147-BCCP (lane 1) or SVV-BCCP (lane 2) proteins were separated on 12% SDS-PAGE, electroblotted onto a PVDF membrane, and then probed with soluble scFv-M6-1B9. The scFv was detected using peroxidase-conjugated mAb anti-HA. The positions of molecular mass markers are shown on the left. **D **CD147 on U937 cells was stained with soluble scFv-M6-1B9 and then probed by mouse anti-HA-biotin. Subsequently, FITC-conjugated sheep anti-mouse immunoglobulins antibody was added. Monoclonal antibody M6-1B9 was used as a control system for detecting CD147 on U937 cells. The immunofluorescence on cells stained with soluble scFv-M6-1B9 (bold line) or mAb M6-1B9 (thin line) is shown. The dashed line represents background fluorescence of negative control mAb. The *y *axis represents the number of events on a linear scale; the *x *axis shows the fluorescence intensity on a logarithmic scale.

The pComb3X-scFv-M6-1B9 was subsequently transformed into *E. coli *HB2151 to produce the soluble scFv antibody. The presence of soluble scFv in the culture supernatant was detected by Western immunoblotting regarding HA and His tags. The reactive bands revealed by anti-HA or anti-His were located at the same molecular weight (~30 kDa) (Figure 3A). This result indicates that soluble scFv-M6-1B9 was successfully produced by *E. coli *HB2151.

The specificity of soluble scFv-M6-1B9 was analyzed by ELISA using CD147-BCCP as antigen (Figure 3B). Various dilutions of soluble scFv-M6-1B9 were represented the positive signal with CD147-BCCP. No signal was detected in the control well of survivin-BCCP (SVV-BCCP) antigen. Subsequently, Western immunoblotting was used to confirm the specificity of the generated scFv-M6-1B9 against recombinant CD147. A specific band of CD147-BCCP at ~35 kDa was detected by probing with soluble scFv-M6-1B9 (Figure 3C). In addition, the native epitope of CD147 on the U937 cell surface was recognized by soluble scFv-M6-1B9 using flow cytometric analysis. The mean fluorescence intensity (MFI) of CD147 cell surface expression on U937 cells stained with soluble scFv-M6-1B9 was 10.42 (Figure 3D). This was similar to the value for the original antibody, M6-1B9, which MFI was 9.21 as shown in Figure 3D. These results strongly suggested that the generated soluble scFv-M6-1B9 carry a CD147-specific paratope which recognized both recombinant and native CD147.

**Figure 4:**
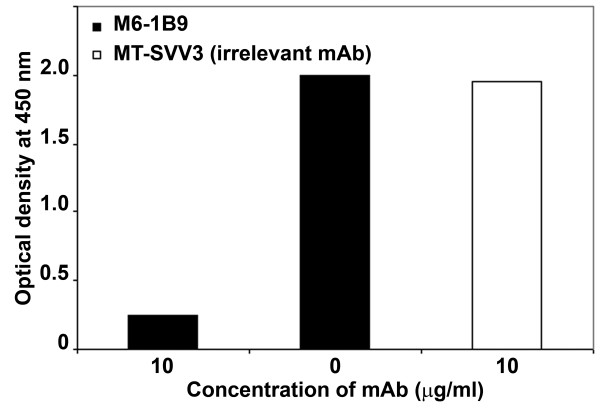
**Competitive binding analysis of soluble scFv-M6-1B9 and mAb M6-1B9**. CD147-BCCP was added onto avidin-coated wells. The mixture contained soluble scFv-M6-1B9 and mAb M6-1B9 or mAb MT-SVV3 at ratio 1:1 was added into the well. The bound scFv was detected by peroxidase-conjugated mAb anti-HA.

To further characterize the specificity of the produced scFv, the inhibiting activity of soluble scFv-M6-1B9 with the original monoclonal antibody, M6-1B9, was tested. The optical density of mixture of soluble scFv-M6-1B9 and mAb M6-1B9 was lower than soluble scFv-M6-1B9 alone (Figure 4). In contrast, the irrelevant mAb MT-SVV3 did not show the inhibition effect. This indicates that the scFv-M6-1B9 recognized the same antigenic determinant as its original mAb M6-1B9.

### Intracellular expression of scFv-M6-1B9 intrabody abated cell surface expression of CD147 on 293A cells

**Figure 5:**
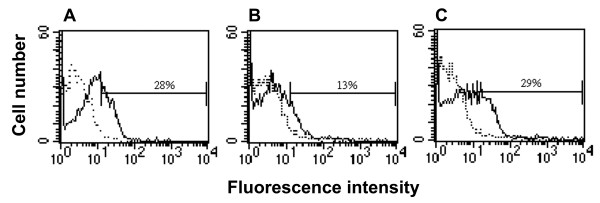
**Inhibition of CD147 surface expression on 293A cells by M6-1B9 intrabody**. 293A cells were transduced with recombinant adenovirus harboring scFv-M6-1B9 or scFv-SVV3. Cell surface staining of CD147 on **A **untransduced and **B **scFv-M6-1B9 or **C **scFv-SVV3 transduced cells was performed using CD147 mAb, M6-1B9 (bold lines) or irrelevant isotype matched mAb (dashed lines). PE-conjugated F(ab')2 fragment of sheep anti-mouse immunoglobulins antibody were used as a secondary antibody. The percentage (%) of CD147 positive cells was indicated.

The compatibility of scFv-M6-1B9 with a eukaryotic expression system was examined by transducing the recombinant adenovirus harboring scFv-M6-1B9 into 293A cells. Alteration of surface expression of CD147 in transduced 293A cells was examined at 36 h after transduction. CD147 cell surface expression was decreased in scFv-M6-1B9 adenovirus-transduced 293A cells compared to untransduced cells (Figure 5). In contrast, no alteration of CD147 expression was observed on scFv-SVV3 adenovirus-transduced cells (Figure 5). This result revealed that intracellular expression of scFv-M6-1B9 as intrabody could diminish CD147 expression on cell surface of intrabody-expressing 293A cells.

### Colocalization of scFv-M6-1B9 intrabody and CD147 in 293A cells

**Figure 6:**
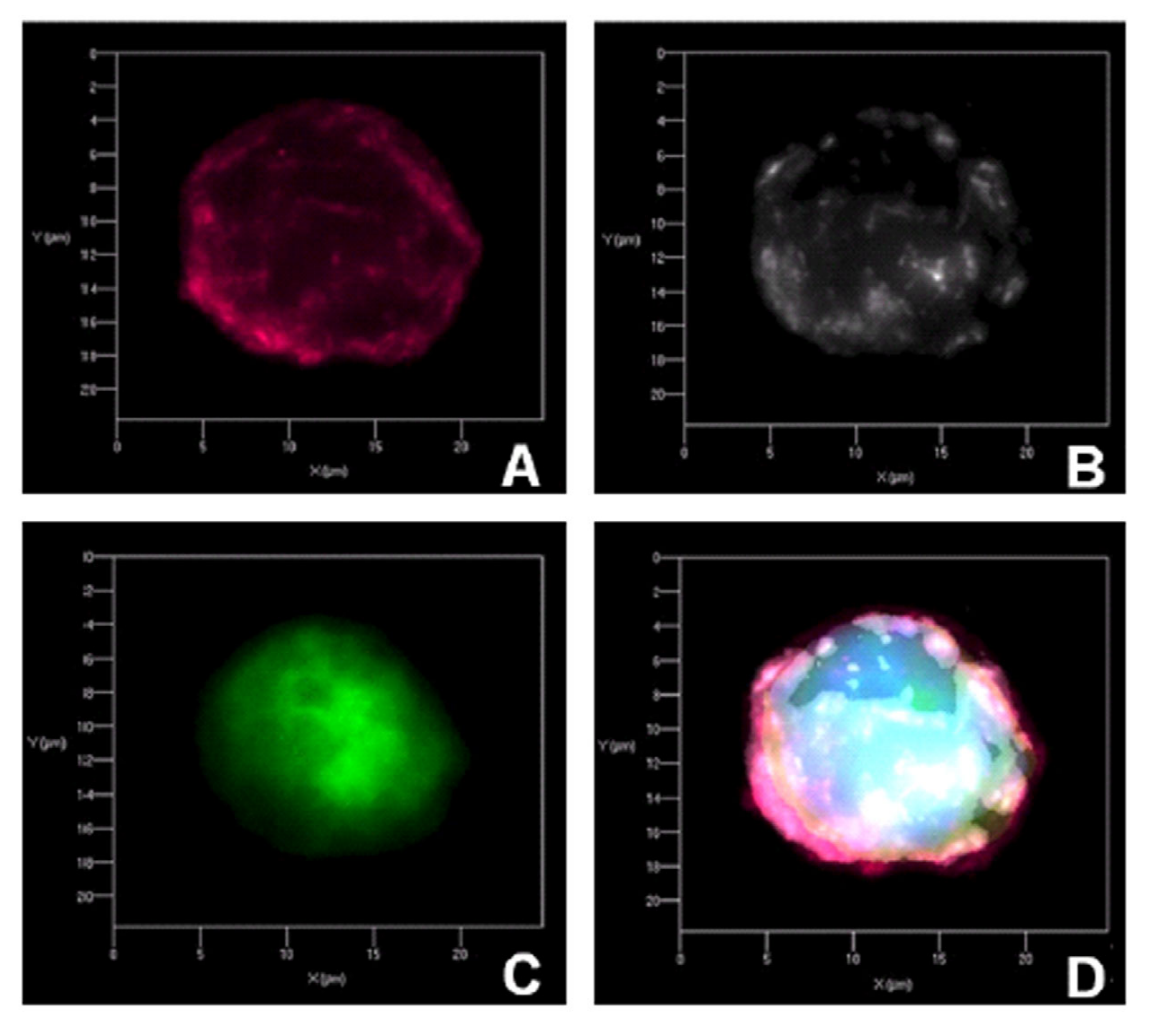
**Immunocytochemical analysis for colocalization of CD147-intrabody**. The transfected 293A cells were fixed and incubated with a mixture of biotinylated anti-human extracellular matrix metalloproteinase inducer (EMMPRIN) mAb and rabbit anti-HA mAb. Then, cells were stained with the mixture of Cy5-conjugated streptavidin and Cy3-conjugated anti-rabbit-IgG mAb. Nuclei were counterstained with DAPI (blue). Three-dimensional (3D) image of the transfected 293A cells was verified. **A **CD147 on transfected 293A cell stained with biotinylated anti-human EMMPRIN mAb (red), **B **scFv-M6-1B9 intrabody in transfected 293A cell stained with rabbit anti-HA mAb (white), **C **GFP positive in transfected 293A cell and **D **overlay. For 3D image of colocalization of CD147-intrabody see additional file.

Colocalization of scFv-M6-1B9 intrabody and CD147 within transfected 293A cells was elucidated. As shown in Figure 6 and in the three-dimensional movies (Additional file 1), scFv-M6-1B9 intrabody was found intracellularly and colocalized with CD147. This result implied that scFv-M6-1B9 protein fused with ER-retention signal was successfully expressed and retained the CD147 molecule inside the cell.

## Discussion

CD147 plays a crucial role in several tissues, but is particularly dense on the surface of activated T-lymphocytes [1,16] and malignant tumor cells [38-40]. Diminishing the expression of CD147 on the cell surface could serve as a step towards exploring the significance of CD147 in cellular functions. In the present study, we successfully employed an intrabody-based approach to reduce the expression of CD147 on the 293A cell surface. This opens new prospects for uncovering the functional role of CD147.

Critical residues implicated in antigen binding from a given antibody paratope are important for the construction of different antibody formats. The deduced amino acid residues responsible for paratope in CDR regions of the scFv-M6-1B9 were identified *via *the WAM algorithm [33] and can be numbered following Kabat's rule [34]. These confirmed the precise cloning of the immunoglobulin variable domains of anti-CD147 mAb, M6-1B9, into the phagemid vector.

Two antibody formats, Fab and scFv, were exploited for antibody phage display. To assess whether Fab-M6-1B9 or scFv-M6-1B9 on gpIII of phage particles have exclusive activities, both antibody formats were generated and evaluated. The expression of phage-displayed scFv-M6-1B9 was significantly greater than phage-displayed Fab-M6-1B9, and both antibody fragments could recognize the CD147 protein. Noticeably, the scFv format exhibited a greater binding activity compared to the Fab format. The expression level of scFvs in *E. coli *is typically higher than Fabs and generates a more efficient antibody display on the phage particle. The interdomain disulfide bond at the C-terminal of the constant region, which plays an important role in Fab stabilization, is predisposed to provide lower production yield than scFvs [41].

Recognition of the amber stop codon between the scFv and gpIII genes that occurs during the expression of pComb3X-scFv-M6-1B9 in the non-suppressing HB2151 *E. coli *strain resulted in the production of soluble scFv-M6-1B9 [42,43]. This antibody fragment was specifically targeted both recombinant CD147 (non-glycosylated form) and native CD147 (glycosylated form) expressed on U937 cell surface. In addition, we demonstrated the inhibition of soluble scFv-M6-1B9 by the original antibody, M6-1B9. These data show that the soluble antibody fragment contained a properly folded, bioactive paratope which recognized both non-glycosylated and glycosylated forms of CD147. CD147 is a highly glycosylated membrane protein. The variation in its molecular weight, ranging between 30 and 66 kDa, arises from different glycosylation patterns [2]. Targeting of both glycosylated and non-glycosylated CD147 molecules *via *scFv-M6-1B9 will be useful as a tool to knockdown the molecules in various cell types.

Generation of adenoviral recombinants carrying scFv-M6-1B9 intrabody in 293A cells was deemed successful, since the cell surface expression of CD147 on these transduced cells declined (Figure 5). It is an open question as to whether this is due to the disruption of CD147 production or the retention of CD147 within the cells. Possibly the ER retention signal, KDEL, sequestered scFv-M6-1B9 intrabody in the ER. This sequestration resulted in the binding of intrabody to the newly synthesized CD147 and retains this molecule inside the cells, as demonstrated by three-dimensional imaging. This result coincided with the functional knockdown of major histocompatibility complex I (MHC-I). Anti-MHC-I intrabody containing this ER retention signal could reduce the surface expression of MHC-I on human umbilical vein endothelial cells (HUVEC) [44]. Thus the KDEL may be necessary for the single-chain antibody protein to diminish the expression of cell surface molecules. The remaining CD147 could be due to the incomplete knockdown effect resulting from transient expression of the scFv-M6-1B9 intrabody.

Intrabodies demonstrate an alternative strategy of gene inactivation that targets genomic DNA or mRNA. Unlike RNAi technology, intrabodies act at the posttranslational level and can be directed to relevant subcellular compartments [27,31,32]. This technique has been employed to diminish expression of a variety cell surface molecules [44-46]. Downregulation of the CD147 surface molecule on 293A cells by means of scFv intrabody expression was achieved in the present study. Nevertheless, RNAi technology has been successfully employed to restrain the expression of CD147 and study the function and mechanism of CD147 in the development of tumor cell lines [25,47]. We therefore aim to introduce the intrabody approach as an alternative method for studying the function and mechanism of CD147 in a variety of metastatic tumors in the near future.

## Conclusion

We provide evidence that intrabody technology can be used to diminish the expression of CD147 on 293A cells. Assessment of the fundamental function of CD147 could be achieved in the near future.

## Methods

### 1. Cell culture

Hybridoma cells producing anti-CD147 mAb, M6-1B9 (isotype IgG3) [8,14] were cultured in Iscove's Modified Dulbecco's medium (Gibco, Grand Island, NY) supplemented with 10% fetal bovine serum (FBS) (Gibco), 40 μg/ml gentamicin and 2.5 μg/ml amphotericin B. An embryonic human kidney cell line (293A) (Invitrogen, Carlsbad, CA) was cultured in Dulbecco's Modified Eagle's medium (DMEM) (Gibco) supplemented with 10 mM non-essential amino acids, 10% FBS, penicillin (100 Units/ml), and streptomycin (100 μg/ml). The human monocytic cell line (U937) was cultured in RPMI 1640 medium (Gibco), penicillin (100 Units/ml), and streptomycin (100 μg/ml). All cells were maintained in a humidified atmosphere of 5% CO_2 _at 37°C.

### 2. *E. coli *strains and vectors

*E. coli *strains, TG-1 and HB2151 were kindly provided by Dr. A.D. Griffiths, MRC, Cambridge, UK. *E. coli *strains, Origami B and XL-1 Blue were purchased from Novagen (Madison, WI) and Stratagene (La Jolla, CA), respectively. The pComb3HSS, pComb3X, modified pAdTrack and pAdEasy vectors were generous gifts from Dr. C.F. Barbas [28,29], The Scripps Research Institute, La Jolla, CA. The pAK400cb vector was a kind gift from Dr. V. Santala (University of Turku, Finland).

### 3. Generation of Fab-M6-1B9 cDNA fragments

Total RNA was extracted from 5 × 10^6 ^M6-1B9-producing hybridoma cells using TRIzol^® ^(Invitrogen) according to the manufacturer's instructions. Complementary DNA (cDNA) was synthesized from 1μg total RNA using specific primers, *i.e*. heavy-chain Fd 3' primer (5'-GGG GGT act agt CTT GGG TAT TCT AGG CTC-3'; the *Spe*I restriction site at 5' overhang designated in small letters) and murineκ light-chain 5' primers (5'-GCG CCG tct aga ATT AAC ACT CAT TCC TGT TGA A-3'; the *Xba*I restriction site at 5' overhang designated in small letters). Resulting first-strand cDNA was used as a template for the amplification of Fab fragments. Specific oligonucleotide primers [42] were used to amplify heavy chain and light chain gene segments *i.e. *heavy-chain variable forward primer (5'-AGG TCC AGC TGc tcg agT CTG G-3'; the *Xho*I restriction site at 5' overhang designated in small letters) and heavy-chain Fd 3' primer; murine light-chain variable forward primer (5'-CCA GTT CCg agc tcg TGA TGA CAC AGT CTC CA-3'; the *Sac*I restriction site at 5' overhang designated in small letters) and murineκ light-chain 3' primers. PCR amplification was performed as formerly described [28].

### 4. Construction of phagemid expressing Fab-M6-1B9

The phagemid expressing Fab-M6-1B9 was constructed. The DNA fragments were digested with *Spe*I/*Xho*I and *Sac*I/*Xba*I, respectively, and then cloned into *Spe*I/*Xho*I and *Sac*I/*Xba*I sites of the phagemid expression vector pComb3HSS. The ligation product was transformed into the competent *E. coli *XL-1 Blue cells. The clones with both inserts were selected on Luria-Bertani (LB) agar containing 100 μg/ml of ampicillin. The plasmid from transformed *E. coli *XL-1 blue was prepared by QIAGEN Miniprep Kit (Qiagen, Hilden, Germany) and digested with *Spe*I/*Xho*I and *Sac*I/*Xba*I. The corrected plasmid was subsequently transformed into *E. coli *TG-1. The transformant bacteria were selected on LB agar containing ampicillin (100 μg/ml). Restriction fragment analysis of the purified plasmid was performed using *Spe*I/*Xho*I and *Sac*I/*Xba*I. The amplified product was checked for an inserted gene in the purified plasmid as described above. The ligated product was named pCom3H-Fab-M6-1B9.

### 5. Conversion of a M6-1B9 specific Fab into a single chain antibody fragment (scFv)

IgG-specific variable heavy (V_H_) and light (V_L_) chain gene fragments from purified pCom3H-Fab-M6-1B9 were amplified using PCR system (Eppendorf, Germany) for 30 cycles in the first round (at 94°C for 15 sec, at 56°C for 30 sec, at 72°C for 90 sec and 10 min at 72°C for final extension), with each forward and backward oligonucleotide primers set, *i.e.*, V_H _fragments used MSCVH14 (5'-GGT GGT TCC TCT AGA TCT TCC CTC GAG GTR AAG CTT CTC GAG TC-3') and MSCG3_B (5'-CCT GGC CGG CCT GGC CAC TAG TGA CAG ATG GGG CTG TTG TTG T-3') primers, V_L _fragments used OmpSeq (5'-AAG ACA GCT ATC GCG ATT GCA G-3') and MSCJK5-BL (5'-GGA AGA TCT AGA GGA ACC ACC CCC ACC ACC GCC CGA GCC ACC GCC ACC AGA GGA TTT CAG CTC CAG CTT GGT CCC-3') primers as described previously [42]. Then, the purified fragments from QIAGEN PCR purification kit (Qiagen) were used as templates for the second round of PCR amplification to extend a linker. The amplified V_H_-linker and V_L_-linker PCR products were combined in a PCR reaction mixture. Twenty cycles (at 94°C for 15 sec, at 56°C for 30 sec, at 72°C for 2 min and final extension for 10 min at the same temperature) were performed. These products were gel-purified, digested with *Sfi*I, cloned into phagemid vector pComb3X and transformed into electrocompetent *E. coli *TG1. The transformed cells were then grown and plated onto LB agar containing ampicillin. Colonies bearing the pComb3X-scFv-M6-1B9 construct were confirmed by *Sfi*I restriction enzyme digestion and PCR. Finally, the inserted gene fragment was sequenced using an ABI 3100 automatic sequencer.

### 6. Preparation of phage-displayed scFv-M6-1B9

A single colony of *E. coli *TG1 harboring pComb3X-scFv-M6-1B9 was chosen from an LB agar plate containing ampicillin for phage-displayed scFv-M6-1B9 preparation as previously described [48]. In brief, transformed bacteria were grown in 2× TY broth containing ampicillin (100 μg/ml) at 37°C with shaking at 200 rpm. The precultured bacteria were subsequently transferred to the same medium containing 1% (w/v) glucose, 1 mM Isopropyl-β-D-thiogalactopyranoside (IPTG) and cultivated at 25°C until the optical density at 600 nm (OD_600_) reach 0.5. After induction, the bacterial culture was further infected with 10^12 ^t.u./ml of VCSM13 helper phages and left at 37°C for 30 min without shaking. The subsequent steps were performed as described previously [48].

### 7. Preparation of plasmid vector encoding CD147Ex-BCCP

A pair of specific primers, CD147*Nde*I (5'-GAG GAG GAG GTc ata tgG CTG CCG GCA CAG TCT TC-3' ; the *Nde*I restriction site at 5' overhang designated in small letters) and CD147*EcoR*I (5'-GAG GAG GAG CTg aat tcG TGG CTG CGC ACG CGG AG-3' ; the *EcoR*I restriction site at 5' overhang designated in small letters), were synthesized in order to amplify CD147 extracellular domain coding sequence from the phagemid pComb8-CD147Ex vector [48] using ProofStart DNA polymerase (Qiagen). The subsequent steps for protein expression using pAK400cb backbone in *E. coli *Origami B strain were performed as described previously [49,50]. The biotinylated CD147Ex-BCCP fusion protein was detected by indirect ELISA using mouse anti-CD147 mAbs including M6-1D4, M6-1B9, M6-1E9, M6-1F3, M6-2B1 and M6-2F9 [8] and peroxidase-conjugated goat-anti-mouse immunoglobulins (KPL, Gaithersburg, MD).

### 8. Immunoassay for phage-displayed scFv-M6-1B9 by ELISA

Microtiter plates (NUNC, Roskilde, Denmark) were coated with 50 μl of 10 μg/ml avidin in carbonate/bicarbonate buffer pH 8.6 overnight at 4°C. The plate was then blocked with 200 μl of 2% skimmed milk in PBS for 1 h at room temperature (RT). The wells were washed five times with 0.05% Tween-20 in PBS. After washing, 50 μl of 100 μg/ml BCCP fusion proteins, *i.e. *CD147-BCCP or SVV-BCCP [50] in 2% skimmed milk, was added and the mixture was incubated for 1 h at RT. Unbound antigen was washed out and 50 μl of phage-displayed scFv-M6-1B9 were added and incubated in a moist chamber for 1 h at RT. The plate was washed thoroughly with 0.05% Tween 20 in PBS 5 times, and peroxidase-conjugated anti-M13 phage mAb (Amersham Pharmacia Biotech, Buckinghamshire, UK) was added to each well. Wells were then washed again prior to adding 100 μl 3,3',5,5'-tetramethyl-benzidine (TMB) substrate. The OD at 450 nm was measured by an ELISA plate reader (TECAN, Austria) after adding 1 N HCl to stop the reaction. mAb M6-1B9 specific for CD147 [8] was used as an antibody control in the ELISA system.

### 9. Preparation of soluble scFv-M6-1B9

Soluble scFv-M6-1B9 was produced by expressing pComb3X-scFv-M6-1B9 phagemid in the non-suppressor *E. coli *strain HB2151. Transformed bacteria were grown in 10 ml of SB broth containing ampicillin (100 μg/ml) at 37°C for 18 h. Ten microlitres of precultured bacteria were subsequently transferred to 10 ml of the same medium containing 1% (w/v) glucose and ampicillin (100 μg/ml), then cultivated at 37°C until the absorption at 600 nm reached 0.5. The precultured bacteria were then transferred to 90 ml of the same medium and cultivated at the same temperature until the OD_600 _reached 1.5. Then, IPTG was added to the culture at a final concentration of 1 mM. After induction, the bacteria were grown at 25°C for 20 h. Cells were centrifuged at 15,000 g for 30 min at 4°C to collect the supernatant (containing extracellular soluble scFv). Protein was precipitated with saturated (NH_4_)_2_SO_4 _in an ice bath and concentrated with Amicon Ultra centrifugal filter units (Millipore, Cork, Ireland). Finally, the concentrated protein was reconstituted with 500 μl of 0.15 M PBS, pH 7.2. ELISA, Western immunoblotting and flow cytometric analysis were performed to detect the antigen-binding affinity of soluble scFv-M6-1B9.

### 10. Binding assay of soluble scFv-M6-1B9 by ELISA

Microtiter plates were coated with 50 μl of 10 μg/ml avidin in carbonate/bicarbonate buffer pH 8.6 overnight at 4°C. The plates were then blocked with 200 μl of 2% bovine serum albumin (BSA) in PBS for 1 h at RT. The wells were washed five times with 0.05% Tween-20 in PBS and 50 μl of 100 μg/ml BCCP fusion proteins [50] in 2% BSA were added and incubated for 1 h at RT. The unbound antigen was washed out. Fifty microlitres of scFv-M6-1B9 at various dilutions were added and incubated in a moist chamber for 1 h at RT. The plates were washed thoroughly with 0.05% Tween 20 in PBS for 5 times and peroxidase-conjugated mAb anti-HA (Roche, Indianapolis, IN) was added to each well. The wells were then washed again prior to adding 100 μl TMB substrate and the OD at 450 nm measured after adding 1 N HCl to stop the reaction. mAb M6-1B9 was used as an antibody control in the ELISA system.

### 11. Competitive binding analysis of soluble scFv-M6-1B9 and mAb M6-1B9

Microtiter plates were coated with 50 μl of 10 μg/ml avidin in carbonate/bicarbonate buffer (pH 8.6) and left overnight at 4°C. The plate was then blocked with 200 μl of 2% BSA in PBS for 1 h at RT. The wells were washed 5 times with 0.05% Tween-20 in PBS and 50 μl of 100 μg/ml BCCP fusion proteins [50] in 2% BSA were added and incubated for 1 h at RT. The unbound antigen was washed out. Fifty microlitres of the mixture containing soluble scFv-M6-1B9 at dilution 1:250 and 20 μg/ml mAb M6-1B9 or mAb against survivin (MT-SVV3) at ratio 1:1 were added. After incubation in a moist chamber for 1 h at RT, the plate was washed thoroughly with 0.05% Tween 20 in PBS for 5 times. Peroxidase-conjugated mAb anti-HA was added to each well. The wells were then washed again prior to adding 100 μl TMB substrate. The OD at 450 nm was measured after adding 1 N HCl to stop the reaction.

### 12. Immunofluorescence analysis of the reactivity of soluble scFv-M6-1B9

U937 cells were adjusted to 1 × 10^7 ^cells/ml with 1% BSA-PBS-NaN_3 _and blocked on ice with human AB serum at the ratio of 1:10 for 30 min. Fifty microlitres of 1:10 dilution in 1% BSA-PBS-NaN_3 _of soluble scFv-M6-1B9 were added to 50 μl of blocked cells and incubated on ice for 30 min. Cells were washed twice with 1% BSA-PBS-NaN_3_. Subsequently, fifty microlitres of 20 μg/ml mouse anti-HA-biotin (Sigma, St Louis, MO) were added and the cells were incubated on ice for 30 min. After washing, cells were resuspended with 20 μl 1% BSA-PBS-NaN_3_. FITC-conjugated sheep anti-mouse immunoglobulins antibody (Chemicon International, Melbourne, Australia) was then added. Cells were incubated on ice for another 30 min. Finally, cells were washed 3 times with 1% BSA-PBS-NaN_3 _and fixed with 1% paraformaldehyde-PBS. Fluorescence reactivity of soluble scFv-M6-1B9 with CD147 on U937 cells was analyzed by flow cytometry.

### 13. Immunoblot analysis

Recombinant phage antibodies were separated on a 12% SDS-PAGE gel under reducing conditions, then transferred onto a polyvinyllidene fluoride (PVDF) membrane. The membranes were blocked with 5% skimmed milk in PBS, and then incubated with mouse anti-gpIII mAb (Exalpha Biologicals, Inc., Watertown, MA). After washing, peroxidase-conjugated goat anti-mouse immunoglobulins antibody were added to the membranes. The peroxidase reaction was visualized using an enhanced chemiluminescent (ECL) substrate detection system (GE Healthcare, Buckinghamshire, UK).

To determine the binding activity of soluble scFv-M6-1B9, BCCP fusion proteins were separated on 12% SDS-PAGE, electroblotted onto PVDF membrane, and then were probed with soluble scFv-M6-1B9 and traced by peroxidase-conjugated mAb anti-HA. The immunoreactive bands were visualized as described previously.

### 14. Assembly of intrabody construct in pAdTrackCMV

The scFv coding regions were flanked by a human κ light chain leader sequence at the 5'-end, and a sequence encoding the HA tag (YPYDVPDYA) and the ER retention signal (KDEL) at the 3'-end. The intrabody coding regions from pComb3X-scFv-M6-1B9 were then excised by digestion with *Sfi*I and cloned into modified pAdTrackCMV [28]. This adapter fragment contains compatible *Sfi*I sites, which were used for cloning the scFv-M6-1B9 intrabody against CD147 into the adenovirus vector. The generation of recombinant adenoviruses was done essentially as previously described [51]. Briefly, 6 × 10^4 ^293A cells in 500 μl DMEM containing 10% FBS and antibiotics were plated on a polystyrene 24-well plate for 24 h before transfection. Transfection mixture was prepared by adding 1 μg pAdE-scFv-M6-1B9 in 50 μl DMEM to 0.25 μl transfectin (Bio-Rad, Hercules, CA) in 50 μl DMEM and followed by incubation at RT for 20 min. The mixture was then added to the cells and incubated at 37°C in 5% CO_2 _for 4 h. Four hundred microlitres of DMEM containing 10% FBS and antibiotics were added into the wells and plates were further incubated at 37°C in 5% CO_2 _for 7 days. High titer viral stocks were produced and purified using a ViraBind™ Adenovirus purification kit (Cell Biolabs, San Diego, CA).

### 15. Flow cytometry analysis for CD147 surface expression

Five hundred microlitres of 1.2 × 10^5 ^cells/ml 293A were transduced with 10 MOI (~80% of the cells were infected) of adenovirus encoding scFv-M6-1B9 intrabody. After 36 h, 293A cells were removed from 24-well tissue culture plates and washed 3 times with PBS. Cells were then blocked with human AB serum for 30 min on ice. Fifty microlitres of 20 μg/ml purified mAb M6-1B9 in 1% BSA-PBS-NaN_3 _were added to 50 μl of blocked cells and incubated on ice for 30 min. Cells were washed twice with 1% BSA-PBS-NaN_3 _and resuspended with 20 μl 1% BSA-PBS-NaN_3_. Subsequently, twenty-five microlitres of PE-conjugated F(ab')_2 _fragment of sheep anti-mouse immunoglobulins antibody were added and incubated on ice for 30 min. Finally, cells were washed 3 times with 1% BSA-PBS-NaN_3 _and fixed with 1% paraformaldehyde-PBS. Fluorescence reactivity of the stained cells was investigated by flow cytometry. Adenovirus encoding scFv specific to survivin (scFv-SVV3) intrabody constructed by the same technique was used as transduction control.

### 16. Immunocytochemical analysis for CD147-intrabody colocalization

For analysis of CD147 and intrabodies on GFP-positive 293A, transfected cells were trypsinized and fixed for 10 min with 3.7% formaldehyde in PBS containing 50 mM MgCl_2_. Fifty microlitres of 1 × 10^6 ^fixed cells were placed on a silane-coated slide and air-dried. Following washing, cells were permeabilized with 0.2% Triton-X 100 for 12 min. Slides were then washed in PBS containing 50 mM MgCl_2 _and blocked with 1% BSA in SSC at RT for 5 min. Then, the fixed cells were incubated with a mixture of biotinylated anti-human extracellular matrix metalloproteinase inducer (EMMPRIN) mAb (0.1 μg/ml; R&D systems, Minneapolis, MN) and rabbit anti-HA mAb (Sigma) at 4°C overnight. After washing, cells were blocked and then incubated with the mixture of Cy5-conjugated streptavidin (Amersham Life Sciences, Inc, Buckinghamshire, UK) and Cy3-conjugated anti-rabbit-IgG mAb (Sigma) at RT for 30 min. Nuclei were counterstained with DAPI. Imaging of stained cells was performed by using a Zeiss Apotome with an AxioCam HRM, AMCA, Cy3, Cy5 and FITC filters in combination with Planapo 63×/1.4 oil objective lens. Images were acquired by using AXIOVISION 4.4 (Carl Zeiss Canada Ltd., Toronto, ON, Canada) in multichanel mode.

### 17. 3D image acquisitions

AXIOVISION 4.4 with deconvolution module and rendering module were used. For every fluorochrome, the 3D image consists of a stack of 80 images with a sampling distance of 200 nm along the *z *and 107 nm in the *xy *direction. The constrained iterative algorithm option was used [52].

## Authors' contributions

KT and NI contributed equally to this work. YY participated in adenoviral vector gene transfer. SM participated in the 3D image analysis. WK participated in the generation of hybridomas, experimental design, co-ordination, and drafted the manuscript. CT established the experimental design, and helped to draft the manuscript. KT, NI, WK and CT wrote the paper. All authors read and approved the final manuscript.

## Supplementary Material

Additional file 1 **Colocalization of CD147 and intrabody**. Colocalization of CD147 (red) and intrabody (white) in the transfected 293A cell was demonstrated in pink. Image acquisition and analysis is as described in Materials and Methods. Click here for file
